# Preliminary Study on a Novel Protocol for Improving Familiarity with a Lower-Limb Robotic Exoskeleton in Able-Bodied, First-Time Users

**DOI:** 10.3389/frobt.2021.785251

**Published:** 2022-01-10

**Authors:** Jan C. L. Lau, Katja Mombaur

**Affiliations:** Canada Excellence Research Chair in Human-Centred Robotics and Machine Intelligence, Systems Design Engineering & Mechanical and Mechatronics Engineering, University of Waterloo, Waterloo, ON, Canada

**Keywords:** lower-limb exoskeletons, wearable robots, familiarization protocol, exoskeleton tutorial, elderly assistance, assistive technology, ageing, able-bodied and first-time healthy users

## Abstract

Lower-limb exoskeletons have been created for different healthcare needs, but no research has been done on developing a proper protocol for users to get accustomed to moving with one. The user manuals provided also do not include such instructions. A pre-test was conducted with the TWIN (IIT), which is a lower-limb exoskeleton made for persons with spinal cord injury. In the pre-test, two healthy, able-bodied graduate students indicated a need for a protocol that can better prepare able-bodied, first-time users to move with an exoskeleton. TWIN was used in this preliminary study and nine users were divided to receive a tutorial or no tutorial before walking with the exoskeleton. Due to COVID-19 regulations, the study could only be performed with healthy, young-to-middle-aged lab members that do not require walking support. The proposed protocol was evaluated with the System Usability Scale, NASA Raw Task Load Index, and two custom surveys. The members who received the tutorial found it easy to follow and helpful, but the tutorial seemed to come at a price of higher perceived mental and physical demands, which could stem from the longer testing duration and the need to constantly recall and apply the things learned from the tutorial. All results presented are preliminary, and it is recommended to include biomechanical analysis and conduct the experiment with more participants in the future. Nonetheless, this proof-of-concept study lays groundwork for future related studies and the protocol will be adjusted, applied, and validated to patients and geriatric users.

## 1 Introduction

### 1.1 State of the Art of Lower-Limb Exoskeletons

Lower-limb exoskeletons have been developed for applications like spinal cord injury (SCI) and stroke rehabilitation. Developed by Ecole Polytechnique Fédérale de Lausanne, the AUTONOMYO is targeted for users with moderate neurological disorders such as Parkinson’s disease, multiple sclerosis, and stroke (Ortlieb et al., 2017). Weighing at 22.5 kg, the exoskeleton has three passive degrees of freedom (DOFs) at the ankle and six active DOFs to enable hip flexion/extension, hip abduction/adduction, and knee flexion/extension. The device provides partial or full assistance at the hips and knees for sit-to-stand, walking, and stair climbing.

Weighing at 25 kg, the TWIN exoskeleton from Istituto Italiano di Tecnologia (IIT) is made for persons with SCI. It has four actuators for flexion/extension at the hips and knees and the device must be used with a set of crutches (Vassallo et al., 2020). The exoskeleton has a modular design with each component coming in three sizes (small, medium, and large) to enable a more customized fit to the user. TWIN can sit, stand, and walk.

The Indego exoskeleton from Parker Hannifin is made for persons with SCI and has a modular design composed of a pelvis, two upper legs, and two lower legs (Tefertiller et al., 2017). It weighs 26 pounds, has four active DOFs for hip and knee flexion/extension, and has two adjustable ankle-foot orthoses. The embedded sensors track the user’s posture and tilt, and the device is used with an external tablet connected via Bluetooth.

Cyberdyne’s HAL lower-limb exoskeleton weighs around 14 kg and has six active DOFs for flexion/extension at the hips, knees, and ankles (Tsukahara et al., 2015). Designed for persons with SCI, users can choose from two types of control. The voluntary control augments the wearer’s joint torque based on the muscle activity estimated with electromyographic (EMG) sensors (Hayashi et al., 2005), whereas autonomous control considers the wearer’s preliminary motion as part of the intention to provide support for a functional motion (Suzuki et al., 2007).

The FDA-approved EksoNR exoskeleton weighs 27 kg and is made for persons with SCI, stroke, and acquired brain injury (Carlan, 2021). With four active DOFs for hip and knee flexion/extension, it provides partial or full assistance for walking and monitors leg movement for adaptive gait training (Contreras-Vidal et al., 2016). The device must be used with a set of crutches.

ReWalk by Argo is another exoskeleton for users with SCI that received an FDA approval (Contreras-Vidal et al., 2016). It has passive spring-loaded ankles and four active DOFs at the hips and knees for flexion/extension. The device can sit, stand, walk, and turn. To take a step when walking, users would shift their body weight forward to trigger the tilt sensor located in the chest strap. Walking aids must be used with the exoskeleton.

The exoskeletons on the market offer different features and functionalities to address specific populations. Although user manuals are provided with the devices, neither of them have a solid protocol in place to help familiarize users how to move with one.

### 1.2 Studies on Exoskeleton Usage and Acceptance

Some research has been done on exoskeleton usage and acceptance. One study trained participants with SCI for 12 weeks and conducted interviews to acquire their expectations and experiences (Manns et al., 2019). A participant commented that it required mental and physical effort to adjust to the exoskeleton’s movements.

Gait impairments associated with aging can affect the quality of life, and the global population of people aged 65 years or above is projected to reach 1.5 billion by 2050 from 703 million in 2019 (United Nations, 2019). A paper evaluated which exoskeletons might be suitable for elderly walking assistance, and concluded that a lot of work still had to be done for these exoskeletons to be appropriate for the elderly (Kapsalyamov et al., 2019). One study investigated the acceptance of lower-limb exoskeletons in geriatric users with reduced motion (del Rio Carral et al., 2021). These elderly participants stated that using such a device would require an ample learning process. Although some believed this technology would enhance autonomy, some were worried that the device would remove it instead. Another study also explored elderly acceptance and perception in robotic assistive devices and concluded that the technologies for geriatric users must be easy and comfortable to use (Shore et al., 2020). Older adults are conscious about their competencies and they maintain it to avoid being alienated from society. Not meeting their needs may cause them to be frustrated, embarrassed, and even abandon the device (Shore et al., 2020).

### 1.3 Purpose of the Presented Preliminary Study

The purpose of the presented preliminary study is to propose a protocol that allows users to familiarize themselves with a lower-limb exoskeleton before and during initial usage. We are using the TWIN, a lower-limb robotic exoskeleton made for persons with SCI and developed by IIT. The necessity for such protocol became obvious when two healthy, able-bodied graduate students experienced difficulty balancing when walking and performing sit-to-stand, had lower back pain, and were startled from the exoskeleton’s predefined gait pattern due to the lack of familiarity in motor interaction. This pre-test led to the assumption that the initial exoskeleton experience would be worse with patients and geriatric users, so we expect that this need exists even more for them to move with one for the first time. The state-of-the-art and qualitative studies on exoskeletons can provide insight towards what could be included in the training, and perhaps what can be changed in the exoskeleton’s predefined trajectories. This paper addresses the first step of meeting this need, which is to develop a protocol only for adult able-bodied, first-time users, and evaluate its effectiveness by analyzing in-person observations and survey responses.

## 2 Methods

### 2.1 Proof-Of-Concept Study and Proposed Protocol

Nine healthy, able-bodied adults volunteered to participate in this proof-of-concept study and were divided to receive the tutorial (T) or no tutorial (NT). Due to COVID-19 restrictions, the population size was small and only lab members were allowed to participate. The average age, height, and mass were 28 years old, 1.698 m, and 66.3 kg respectively. There were four men and one woman for NT, and there were two men and two women for T, who received tutorial instructions in the form of video clips and live demos. They also had the opportunity to practice each exercise during the tutorial at their own pace.

The proposed protocol contains four parts: Preparation, Tutorial, Exo Session, and Ending. NT and T underwent Preparation, Exo Session, and Ending, but only T experienced the Tutorial.

1 Preparation
  a. Invite member to complete a pre-study survey.
  b. Adjust the forearm crutches appropriate to the member.
  c. Obtain member’s stature, mass, lower limb segment lengths, and lower limb segment masses.
  d. Create a new profile of the member on the TWIN tablet.
2 Tutorial
  a. Perform crutches exercises without exoskeleton.
    (1) Bring crutches backwards while sitting.
    (2) Perform sit-to-stand and stand-to-sit with crutches.
    (3) Walk around with crutches.
    (4) Repeat (3) but shift weight to stationary leg for each step.
    (5) Practice turning by pivoting.
  b. Demonstrate TWIN’s sit-to-stand, stand-to-sit, and walking motions without member wearing it.
  c. Don TWIN on member and attach TWIN to ceiling lift via straps.
  d. Lift member slightly off the ground with the ceiling lift.
  e. Activate walking motion to familiarize member with TWIN’s gait pattern without the risk of falling.
3 Exo Session
  a. Perform sit-to-stand and stand-to-sit wearing TWIN.
  b. Walk in TWIN while being supported by a lift (Guldmann GH3 Ceiling Hoist).
    (1) Manual walk mode for 7 m.
    (2) Automatic walk mode for 7 m.
  c. Walk in TWIN while being supported by a person.
    (1) Manual walk mode for 7 m.
    (2) Automatic walk mode for 7 m.
4 Ending
  a. Doff TWIN from member.
  b. Member completes post-study survey, SUS, and RTLX.

### 2.2 Equipment

**FIGURE 1:**
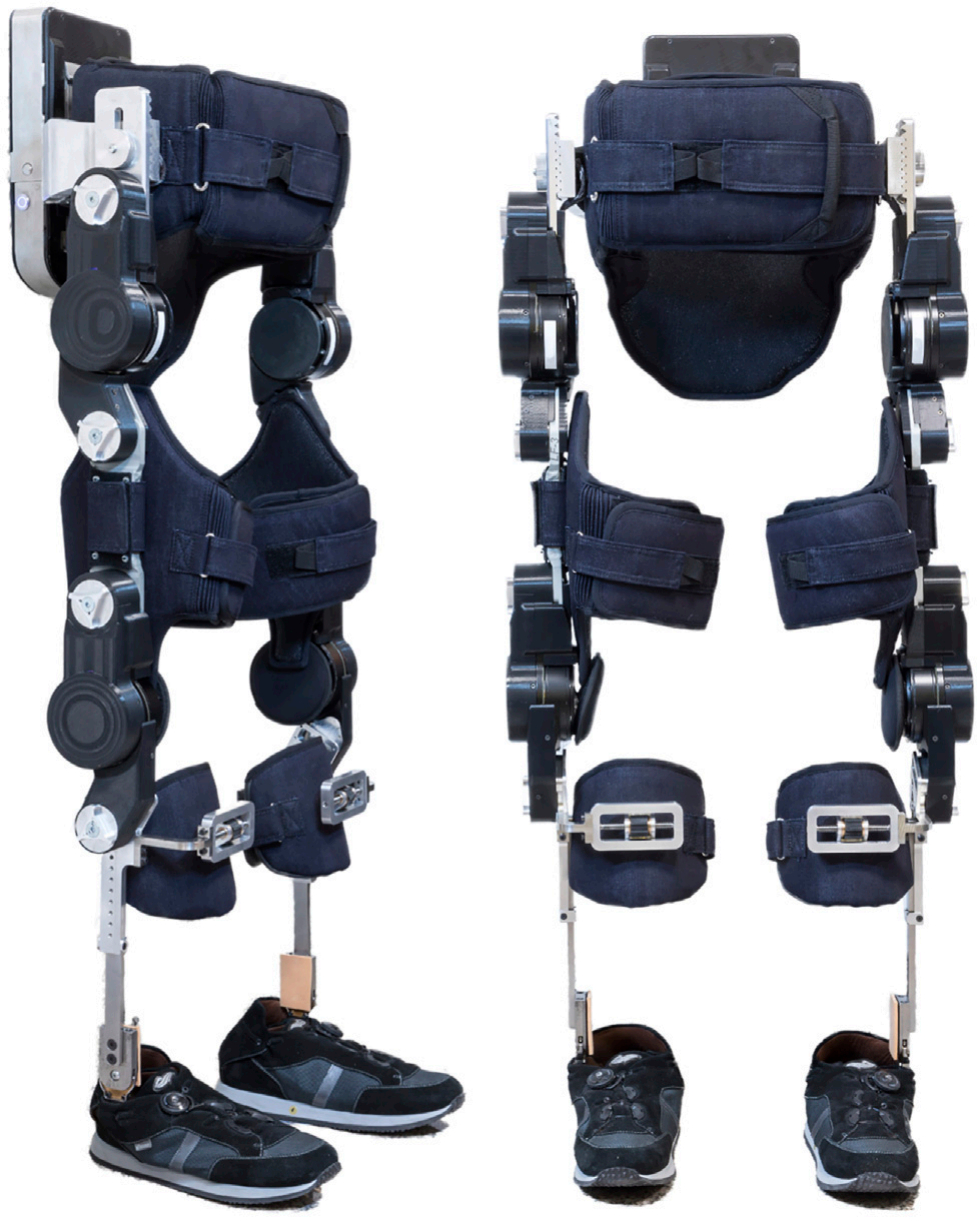
Side and front views of the IIT TWIN exoskeleton.

As mentioned, the exoskeleton used in this preliminary study is IIT’s TWIN. It comes with four walking modes: two impose a predefined gait trajectory on the wearer, and two provide partial or full assistance to the movements imposed by the wearer. For this proof-of-concept study, the latter two modes were not included since the protocol is intended for improving familiarity with the exoskeleton’s predefined gait pattern. The modes used in this study were manual walk mode (MWM) and automatic walk mode (AWM). MWM means each step is triggered manually via the TWIN tablet, and AWM means each step is triggered by the wearer’s forward incline. There is an on-board inertial measurement unit (IMU) located at the back of the pelvis to calculate inclination angles. Figure 1 depicts the TWIN (pictures obtained from IIT’s website). The times for walking, stand-up, and sit-down are set as default by IIT to 2 s, 4 s, and 4 s respectively.

### 2.3 Analysis

System Usability Scale (SUS), NASA Raw Task Load Index (RTLX), and a set of two custom surveys were utilized for measuring TWIN’s usability, member’s perceived cognitive workload, and member’s comfort level respectively. The selection of these questionnaires was inspired by the ones used for evaluating the don/doff procedure of a wrist exoskeleton (Lambelet et al., 2020).

The two custom surveys created were completed before and after the exoskeleton usage to capture the user’s comfort level. The survey statements were slightly different between NT and T, such that T’s included statements about the tutorial received. The statements on the custom surveys can be viewed in the Supplementary Material. Members would respond to the statements on a scale from “strongly agree” to “strongly disagree,” and the results were treated as subjective opinions since Likert scales can be problematic when applying statistical analyses (Bishop and Herron, 2015).

SUS was used in this study to see if the protocol introduced would have any effect on the exoskeleton’s usability. It is an established tool for evaluating the usability of a device (Bangor et al., 2008). The survey consists of ten questions with five response options ranging from “strongly agree” to “strongly disagree.” The final score ranges from 0 to 100, but is not to be interpreted as percentages (Brooke, 2013).

RTLX was used for evaluating sit-to-stand, stand-to-sit, turning, MWM, and AWM. It measures a person’s perceived cognitive workload when performing a task, and the workload aspects are mental demand, physical demand, temporal demand, performance, effort, and frustration (Hart, 2006). These six aspects were each ranked on a scale with 20 equal intervals and were later converted to a final score ranging from 0 to 100 following the RTLX scoring guidelines. The full TLX version takes different weightings for each aspect, but this step was omitted in this proof-of-concept study for simplicity, meaning the aspects shared equal weights (Hart, 2006). A higher score means the person finds the task to be more demanding, they were less successful in accomplishing what they were asked to do, they had to exert higher levels of effort to accomplish their level of performance, or they experienced higher levels of frustration. Instructions on calculating the scores for SUS and RTLX are listed below (Hart, 1986; Lewis, 2018).

SUS Scoring:

1. Convert Likert scale options to values from 0 to 4.
  a. “Strongly Agree” = 4
  b. “Agree” = 3
  c. “Neutral” = 2
  d. “Disagree” = 1
  e. “Strongly Disagree” = 0
2. Sum scores from all odd-numbered questions and subtract 5.
3. Sum scores from all even-numbered questions and subtract from 25.
4. Add adjusted scores from steps 2 and 3, then multiply by 2.5 to obtain the SUS score.
5. Repeat the process for the remaining participants.
6. Calculate the mean SUS score per group by taking the average of the values in the corresponding group.

RTLX Scoring:

1. Count the number of lines from left to right (1–21) marked by the participant.
2. Subtract 1 from the marked line and multiply by 5.
3. Repeat steps 1 and 2 for the other five aspects.
4. Repeat step 3 for all tasks evaluated and the remaining participants.
5. Calculate the mean score for each aspect per group by taking the average of all the scores in the corresponding aspect per group.
6. Calculate the average workload score by averaging the mean scores from the six aspects per group.

For SUS and RTLX, scores were compared between the members who received and did not receive the protocol. It is important to note that outliers in small datasets can skew the overall performance, so the findings should be handled carefully. Due to the small sample size, statistical analysis was omitted in this preliminary study and no statements on significant differences were made regarding the scores. As mentioned earlier, COVID-19 restrictions limited the recruitment size and demographic to only healthy lab members.

## 3 Results

### 3.1 General Observations

Most users struggled to keep themselves upright with the crutches when performing the first sit-to-stand, though their performance seemed smoother when doing stand-to-sit. In general, T managed to turn easier with the TWIN than NT. Per visual observations, most members started off with some difficulties walking, but they eventually adapted to the exoskeleton’s movements throughout the test at different rates. Meanwhile, most users had difficulty triggering AWM steps. With the IMU located at the back of the pelvis, tilting the torso was insufficient to trigger AWM steps despite having a proper fit. They had to tilt forward by the ankles to activate the steps, but the movement felt unnatural.

One NT members’ performance introduced a few safety concerns. He held the crutches with both arms rotated internally while walking, which almost pinched his index finger between the exoskeleton frame and crutch handle twice. Another safety concern was related to his posture because his back was severely hunched forward when walking. He also had large lateral deviations when walking, thereby resulting in an unstable gait.

On the other hand, there was another NT member who naturally outperformed all users, including the ones who received the tutorial. He was the only person who managed to perform sit-to-stand without losing balance on the first try. He only briefly struggled with balancing at the start of the testing after standing up from sitting.

### 3.2 SUS Score

**FIGURE 2:**
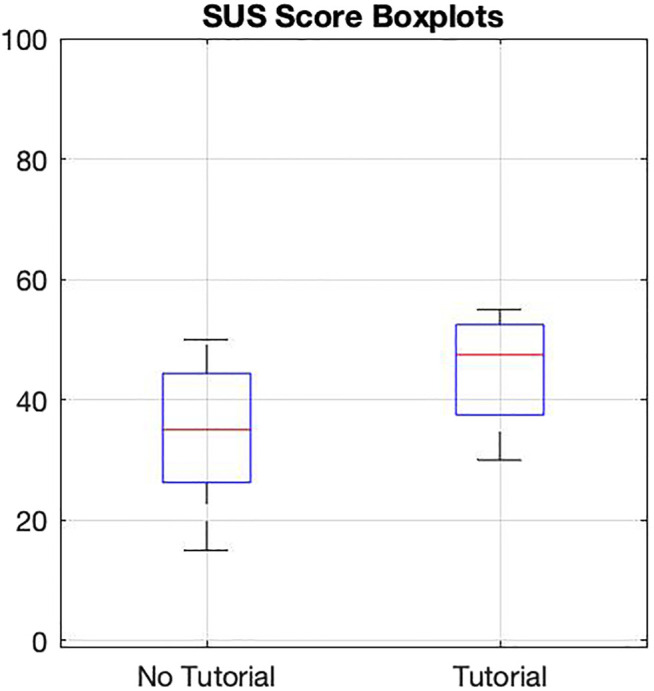
SUS scores of users receiving tutorial and those who did not.

The mean and median usability scores in NT are 34.5 and 35 respectively, with a standard deviation (SD) of 13.3 and an interquartile range (IQR) of 18.125. T has mean and median usability scores of 45 and 47.5 respectively, an IQR of 15, and SD of 10.8. Figure 2 shows the spread of the scores in both groups. The bottom and top lines of each box represent the first and third quartiles, and the red line represents the median. The top and bottom error bars in each boxplot extend to maximum and minimum values that are within one standard error.

### 3.3 RTLX Score

Each member completed five RTLX questionnaires, one for each task. Unlike the SUS, the RTLX does not have a threshold for a demand considered “too high,” hence only a relative comparison between conditions and/or within subjects could be made (Hart, 2006). Table 1 summarizes the mean RTLX scores in NT and T for the five tasks.

**TABLE 1:** Mean RTLX scores for the five tasks of users receiving tutorial and those who did not.

**Sit-to-stand**	**NT**	**T**
Mental Demand	36	31.25
Physical Demand	66	45
Temporal Demand	35	27.5
Performance	35.5	41.25
Effort	58.5	33.75
Frustration	32.5	25
Average Workload Score	43.916 7	33.958 3
**Stand-to-sit**	**NT**	**T**
Mental Demand	20	52.5
Physical Demand	25	50
Temporal Demand	40	32.5
Performance	24.5	35
Effort	22.5	40
Frustration	26	33.75
Average Workload Score	26.333 3	40.625 0
**Turning**	**NT**	**T**
Mental Demand	28.5	51.25
Physical Demand	66	52.5
Temporal Demand	29.5	35
Performance	27	36.25
Effort	67	55
Frustration	17	48.75
Average Workload Score	39.166 7	46.458 3
**MWM**	**N** **T**	**T**
Mental Demand	35	71.25
Physical Demand	56.5	77.5
Temporal Demand	25.5	51.25
Performance	38.5	58.75
Effort	69	77.5
Frustration	35.5	66.25
Average Workload Score	43.333 3	67.083 3
**AWM**	**NT**	**T**
Mental Demand	46.5	67.5
Physical Demand	58.5	65
Temporal Demand	33	47.5
Performance	34.5	50
Effort	59	76.25
Frustration	34	56.25
Average Workload Score	44.250 0	60.416 7

For the sit-to-stand task, T’s average workload score is 22.7*%* less than NT’s. T found sit-to-stand to be mentally, physically, and temporally less demanding. They also did not have to work as hard and were less frustrated when completing the task.

As for the stand-to-sit task, T’s average workload score is 54.3*%* higher than NT’s. T found stand-to-sit to be temporally less demanding, but mentally and physically more demanding. They also had to exert more effort and were more frustrated with the task.

T’s average workload score for turning is 18.6*%* higher than NT’s. Although T thought turning with the exoskeleton was physically less demanding and required less effort, they experienced higher mental and temporal demands and higher frustration.

T’s scores across all aspects of the MWM and AWM tasks are consistently higher than NT’s; their average workload scores in MWM and AWM are 54.8% and 36.5*%* higher than NT’s respectively. They found both walking tasks to be more rushed and demanding (mentally and physically), experienced higher levels of frustration, worked harder to accomplish their level of performance, and found themselves less successful in accomplishing what they were asked to do.

Across all tasks, T had a higher performance score than NT, meaning they perceived themselves as less successful in accomplishing what they were asked to do.

### 3.4 Custom Surveys

All T members agreed that receiving a tutorial on how to use the exoskeleton would be helpful for wearing the TWIN for the first time (two responded with “strongly agree” to this statement). Four NT members agreed that having a tutorial on getting acquainted with the TWIN would better prepare their first time wearing it (one stated “strongly agree”), but one member disagreed with this statement.

T members liked the idea of having a tutorial to prepare themselves for the exoskeleton (three stated “strongly agree” and one stated “agree”). They also agreed that the tutorial helped preparing them for the TWIN (two stated “strongly agree”) and the tutorial was easy to follow (two stated “strongly agree”). One member said “neutral” and three members said “disagree” when asked if the tutorial should be improved before they wear the exoskeleton in the future.

## 4 Discussion

The preliminary study involving the novel protocol has yielded interesting findings that will be validated in a future study with larger groups of external participants. As mentioned earlier, findings from small sample sizes should be handled with care since outliers can influence the overall behavior. The rationale for including the SUS is not to evaluate the usability of the TWIN, but rather to see if receiving the tutorial would affect its usability. T’s average SUS score is 30.4*%* higher than NT’s, meaning the tutorial had some improvement towards the exoskeleton’s usability after one session. Although the average scores fall within the “poor usability” spectrum of the scoring scale (Bangor et al., 2008), it is not a surprise. These are people that do not need an exoskeleton and it usually requires multiple training sessions to learn how to properly walk with one. Since this is the first time the lab members have ever worn such a device and they only had one session, the lower scores were expected.

One clear trend was T’s higher RTLX scores in all aspects of the MWM and AWM tasks. In the tutorial, the T members walked with crutches without wearing the device at their own pace. During both walking tasks, however, the leg swing in the exoskeleton’s predefined trajectory moved at a faster pace, which explains why they felt more rushed and therefore a higher temporal demand score. The higher mental demand score stemmed from the fact that they were constantly recalling what they learned from the tutorial. The higher physical demand score could be influenced by the longer testing duration because the T members received the tutorial immediately before walking in the exoskeleton. With these factors combined, T had to work harder to accomplish their level of performance and were more frustrated. That said, the average workload score in MWM is higher than AWM’s. It could be because of the test’s task sequence: the members experienced MWM before AWM. By the time they performed the AWM task, they had a better idea of what to do and how to walk with the exoskeleton. The order was not randomized because there were too few users to modulate conditions and would otherwise make the data harder to interpret.

Another clear trend was T perceiving themselves as less successful in accomplishing what they were asked to do in all five tasks. It is possible that the tutorial unintentionally created higher performance expectations. Since the members received a tutorial, they could be under the impression that they should be struggling less or not struggling at all, thereby thinking they did not perform as well.

Sit-to-stand results could potentially mean that the tutorial was helpful for performing this task, but the stand-to-sit results suggests that the tutorial only helped users to be more familiar with the pace of it. The reason for these findings is unknown since the members did not comment on it and no conclusions could be drawn from in-person observations. For the turning task, the tutorial was helpful in terms of teaching users the proper technique to turn with an exoskeleton. Similar to the stand-to-sit task, the phenomena observed in the turning task could not be fully explained.

According to the custom surveys, there was a NT member who disagreed that having a tutorial on getting acquainted with the TWIN would better prepare his first time wearing it. This is because he naturally performed well when completing various tasks with the TWIN. People behave differently when interacting with a new device, so it was not a surprise that all lab members had varying levels of adaptation when using the exoskeleton for the first time. Meanwhile, the subjective opinions from T members strongly indicated that the proposed tutorial was useful.

All results presented are preliminary. The protocol has some improvement towards the usability of the exoskeleton and was deemed beneficial by the members who received it, but the workload scores for the walking tasks contradict these results and do not fully prove that the protocol was indeed effective. That said, although performance safety concerns were only observed in one NT member, receiving the proposed protocol could mitigate these safety hazards because it includes a tutorial on using crutches and shifting body weight when walking. Some phenomena observed could not be fully explained due to limited information, so it is recommended to incorporate EMG, motion capture, instrumented crutches, and pressure sensors/force plates for future related studies. Survey results and in-person observations coupled with biomechanical analysis could provide a more complete perspective on the effectiveness of the protocol and enable a deeper investigation on the perceived workload. With a larger sample size, the effects of outlier data could be reduced and statistical analysis could be included for a more in-depth analysis. As mentioned in the Methods section, RTLX does not have a threshold for a demand considered “too high.” One suggestion for utilizing the RTLX is to also evaluate the users performing the same tasks without an exoskeleton to observe the change in perceived workload. Nevertheless, this proof-of-concept study lays groundwork for future related studies, and the protocol will be adjusted to patients and geriatric users.

## 5 Conclusion

Based on the preliminary results, the proposed protocol was beneficial for improving familiarity with a lower-limb robotic exoskeleton in able-bodied, first-time users, but it seemed to come at a price of poorer perceived performance and higher perceived workload. The tutorial had some improvement on the usability of the TWIN exoskeleton after only one session. While one NT member outperformed all users, another NT member exhibited safety concerns related to crutches usage, walking posture, and balance issues. Only healthy lab members could be invited due to COVID-19 restrictions, but this preliminary study justifies the need for a protocol and the proposed tutorial shows potential for improving the familiarity with a lower-limb robotic exoskeleton in able-bodied, first-time users. Next steps include performing a full study with larger groups of external participants, incorporating sensors and instrumented crutches for biomechanical analysis, adjusting the protocol to patients and geriatric users, and increasing the sample size of potential users/patients.

## Data Availability

The datasets presented in this article are not readily available because the volunteers have not given consent for their data to be shared.
